# Role of phosphatase of regenerating liver 1 (PRL1) in spermatogenesis

**DOI:** 10.1038/srep34211

**Published:** 2016-09-26

**Authors:** Yunpeng Bai, Hong-Ming Zhou, Lujuan Zhang, Yuanshu Dong, Qi Zeng, Weinian Shou, Zhong-Yin Zhang

**Affiliations:** 1 Departments of Medicinal Chemistry and Molecular Pharmacology and of Chemistry, Purdue Center for Cancer Research, and Purdue Center for Drug Discovery, Purdue University, 575 Stadium Mall Drive, West Lafayette, IN 47907, USA; 2 Department of Biochemistry and Molecular Biology, Indianapolis, IN 46202, USA; 3 Institute of Molecular and Cell Biology, A*STAR (Agency for Science, Technology and Research), 61 Biopolis Drive, Proteos, Singapore 138673, Republic of Singapore; 4 Department of Pediatrics, Indiana University School of Medicine, Indianapolis, IN 46202, USA

## Abstract

The PRL phosphatases are oncogenic when overexpressed but their *in vivo* biological function is less well understood. Previous gene deletion study revealed a role for PRL2 in spermatogenesis. We report here the first knockout mice lacking PRL1, the most related homolog of PRL2. We found that loss of PRL1 does not affect spermatogenesis and reproductive ability of male mice, likely due to functional compensation by the relatively higher expression of PRL2 in the testes. However, PRL1^−/−^/PRL2^+/−^ male mice show testicular atrophy phenotype similar to PRL2^−/−^ mice. More strikingly, deletion of one PRL1 allele in PRL2^−/−^ male mice causes complete infertility. Mechanistically, the total level of PRL1 and PRL2 is negatively correlated with the PTEN protein level in the testis and PRL1^+/−^/PRL2^−/−^ mice have the highest level of PTEN, leading to attenuated Akt activation and increased germ cell apoptosis, effectively halting spermatozoa production. These results provide the first evidence that in addition to PRL2, PRL1 is also required for spermatogenesis by downregulating PTEN and promoting Akt signaling. The ability of the PRLs to suppress PTEN expression underscores the biochemical basis for their oncogenic potential.

The Phosphatases of Regenerating Liver (PRL1, 2 and 3) constitute a novel class of prenylated phosphatases within the protein tyrosine phosphatase superfamily^1^,^2^,^3^. PRL1 was initially identified as an immediate early gene during liver regeneration^1^. PRL2 and PRL3 were subsequently discovered by their sequence similarity to PRL1^4^. PRL1 and PRL2 are most similar in amino acid sequence, exhibiting 87% identity, while PRL3 is 76% and 79% identical to PRL1 and PRL2, respectively^4^,^5^. Among the three PRL isoforms, PRL1 and PRL2 are expressed ubiquitously across all tissues with PRL1 levels being slightly more restricted and less abundant than PRL2^4^,^6^,^7^,^8^. PRL3 was initially found primarily in heart and skeletal muscle^4^,^6^, although a recent study revealed a more broad but low expression pattern for PRL3^9^.

Unlike other protein phosphatases that function to antagonize the action of protein kinases, PRLs play a positive role in cell signaling and rank among the most oncogenic phosphatases^5^,^10^. PRLs are overexpressed in many tumor cell lines, and cells expressing high levels of PRLs exhibit increased proliferation, migration and anchorage-independent growth^1^,^2^,^11^,^12^,^13^,^14^. Results from *in vitro* studies indicate that PRLs promote cell proliferation and migration through activation of several signaling pathways, such as the Rho family of small GTPases, Src, ERK1/2, and PI3K^14^,^15^,^16^,^17^,^18^. Clinically, up-regulation of PRLs has been associated with many types of advanced stage tumors as well as metastatic lesions and elevated PRL expression is often correlated with increased tumor invasiveness and poor prognosis^5^,^19^. However, in spite of the strong cancer connection, the roles of PRLs in normal physiology are not well understood. To begin to uncover the physiological significance of the PRL phosphatases, both PRL2 and PRL3 knockout mice have been generated. Deletion of PRL2, the most ubiquitously and abundantly expressed PRL family member, leads to growth retardation in both embryos and adult mice and PRL2^−/−^ mice display placenta insufficiency^20^, impairment of spermatogenesis^21^, and defects in hematopoietic stem cell self-renewal^22^. Biochemically, all three phenotypes associated with PRL2 deletion are caused by decreased Akt phosphorylation as a result of up-regulation of the tumor suppressor PTEN. Intriguingly, a role for PRL2 in modulating intracellular Mg^2+^ level through Mg^2+^ transporter CNNMs was also proposed^23^. Interestingly, recent studies suggested that CNNMs may also play a role in reproduction and sperm function^24^,^25^. In contrast, mice deficient for PRL3 were grossly normal, although PRL3-null mice treated with carcinogenic azoxymethane and dextran sodium sulfate developed 50% fewer colon tumors than the wild-type, corroborating a potential role of PRL3 in colon tumorigenesis^9^,^26^. Nothing is known about the physiological function of PRL1 due in part to the absence of informative animal models.

Here, we describe the creation and characterization of PRL1 knockout mice. In contrast to what has been reported for the PRL2^−/−^ mutant animals, PRL1 deficient mice developed normally and did not exhibit any obvious phenotypic alteration. To further investigate the role of PRL1 *in vivo*, we sought to generate mice lacking both PRL1 and PRL2. This was based on the fact that PRL1 is more closely related to PRL2 and shares similar tissue distribution with PRL2. Unfortunately, PRL1/PRL2 double knockout mice were embryonic lethal, indicating a critical requirement for both PRL1 and PRL2 in mouse embryonic development. However, PRL1^−/−^/PRL2^+/−^ and PRL1^+/−^/PRL2^−/−^ mice are viable, which enabled us to interrogate the function of PRL1 in spermatogenesis. We report that the testicular atrophy previously observed in PRL2^−/−^ mice is also found in PRL1^−/−^/PRL2^+/−^ mice, and this phenotype is further aggravated in PRL1^+/−^/PRL2^−/−^ mice. Mechanistically, the testis of PRL1^+/−^/PRL2^−/−^ mice has the highest level of PTEN, leading to attenuated Akt activation and increased germ cell apoptosis. These results provide the first evidence that in addition to PRL2, PRL1 is also required for spermatogenesis by regulating the PTEN/Akt signaling pathway, and support the notion that there is a functional redundancy between PRL1 and PRL2, leading to a “PRL1/2-dose”-dependent phenotype *in vivo*.

## Results and Discussion

### Creation of PRL1 mutant mice

To begin to delineate the physiological function of PRL1, we generated PRL1 knockout mice using a gene trap ES cell clone (CC0606) containing an insertional mutation in the mouse PRL1 locus from Mutant Mouse Regional Resource Centers (MMRRC)^20^. The gene trap vector containing a reporter gene β-galactosidase/neomycin (β-Geo) ORF flanked by an upstream splice-acceptor sequence and a downstream premature polyadenylation signal was inserted into the exon 2 of PRL1 (Figure S1A), which should abrogate transcription of PRL1 exons 2 to 6. Since the insertion site is upstream of the translation start codon for PRL1 within exon 2, the insertion of the gene trap vector would completely eliminate endogenous PRL1 expression. The offspring of the PRL1 gene trapped chimera were confirmed for successful germline transmission as evidenced by genotyping with the heterozygous alleles. Breeding the F1 heterozygous mice gave offspring with all three genotypes including PRL1 wild-type, heterozygous and homozygous deletion animals (Figure S1C).

To confirm PRL1 gene deletion, we first examined PRL1 at the mRNA level by quantitative RT-PCR. Total mRNAs were extracted from the thymus of either wild-type, PRL1-null or PRL2-null mice to confirm the deletion of PRL1 or PRL2. As shown in Figure S2A, PRL1 or PRL2 mRNA was not detectable in the knockout samples. Deletion of PRL1 or PRL2 protein was also confirmed by Western blot analysis using a PRL1/2 antibody, an antibody that recognizes both PRL1 and PRL2^20^,^21^. To evaluate the affinity and specificity of the PRL1/2 antibody, we measured its ability to detect recombinant PRL1, PRL2 and PRL3 proteins (Figure S2B). The results showed that this antibody binds PRL1 and PRL2 with the same affinity but its affinity for PRL3 is at least 100-fold lower. Since PRL2 is 6 amino acids shorter than PRL1, the PRL1/2 antibody can be used to distinguish endogenous PRL1 and PRL2 based on their difference in size. This allows assessment of the relative levels of PRL1 and PRL2 in tissues as well as potential changes in PRL2 levels upon PRL1 deletion. Therefore, tissue lysates from lung and spleen of either wild-type, PRL1^−/−^ or PRL2^−/−^ mice were surveyed for the presence of PRL1 and PRL2 using the PRL1/2 antibody. While endogenous PRL1 and PRL2 are clearly detectable in the wild-type samples, they are absent in those from the respective PRL1-null and PRL2-null mice (Figure S2C). Taken together, these results confirm PRL1 deletion with the “gene-trapped” approach.

### Phenotypic analysis of PRL1 knockout mice

To characterize the phenotype of mice lacking PRL1, we first examined the birth rate of the PRL1 deficient mice by analyzing nearly 700 pups produced by heterozygous mating. Although all possible genotypes were observed in the resulting offspring, the segregation of the PRL1 knockout alleles did not exactly follow the expected Mendelian ratio of 1:2:1 (Table 1). Among the 683 and 519 pups produced by PRL1 and PRL2 heterozygous mating, only 143 and 73 animals were identified as knockouts. These numbers were significantly less than what would be expected for a normal Mendelian distribution of 170 and 130, respectively, for PRL1^−/−^ and PRL2^−/−^. This observation suggests that deletion of either PRL1 or PRL2 presents a survival disadvantage to the animal.

We then measured the body weight of more than 20 males and females with different genotypes at weaning (4 weeks old). In contrast to PRL2^−/−^ mice, which exhibit a 20% decreased body weight in comparison to their wild-type counterparts^20^, the body weight of PRL1^−/−^ mice was not significantly different from that of the wild-type (Figure S3A,B). As reported previously^21^, testis from PRL2^−/−^ males was significantly smaller even when normalized to the whole body weight (Figure S3C). However, a similar organ/whole body weight ratio was observed for both PRL1^−/−^ mutant and wild-type controls (Figure S3C,D). Histological analysis was performed on tissue sections by hematoxylin and eosin staining, and no significant abnormalities were observed in all genotypes (Figure S3E). These data indicate that deletion of PRL1 does not alter overall mouse phenotypes.

### Generation and characterization of PRL1^−/−^/PRL2^+/−^ and PRL1^+/−^/PRL2^−/−^ mice

Given the lack of apparent phenotype for the PRL1^−/−^ mice, we suspected that there might be functional redundancy among the PRLs. Since PRL1 is most homologous to PRL2 (87% amino acid sequence identity) and exhibits a similar expression pattern to that of PRL2, we sought to generate mice lacking both PRL1 and PRL2 in order to assess whether the two phosphatases play similar roles in signaling. Mice heterozygous for both PRL1 (Figure S1A,C) and PRL2 (Figure S1B,C) were intercrossed to generate PRL1^−/−^/PRL2^−/−^ double knockout mice. However, after analysis of more than 300 pups from the PRL1^+/−^/PRL2^+/−^ cross, none of the offspring were found to be PRL1^−/−^/PRL2^−/−^ double knockouts (Table 2). This result suggests that mice lacking both PRL1 and PRL2 are embryonic lethal. To investigate at which stage the embryos were dead, we carried out time mating to harvest embryos at E17.5, E14.5, E11.5 and E9.5 days. Unfortunately, we could not identify any viable double knockout mice at these stages, indicating early embryonic lethality in the absence of both PRL1 and PRL2.

**Figure 1:**
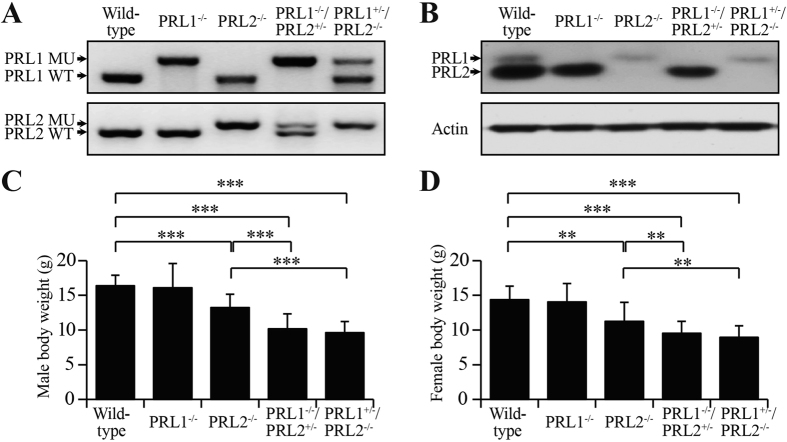
Generation of PRL1^−/−^/PRL2^+/−^ and PRL1^+/−^/PRL2^−/−^ mice. (**A)** PCR strategy for genotyping using mouse tail DNA. (**B)** Endogenous PRL1 and PRL2 protein products were determined by Western blot on lung lysates from different genotypes. (**C,D)** Body weights of 4-weeks-old male mice (**C**) or female mice (**D**) with different genotypes were determined. Body weights of PRL2^−/−^, PRL1^−/−^/PRL2^+/−^ and PRL1^+/−^/PRL2^−/−^ males and females were significantly less than those of wild-type and PRL1^−/−^ males and females. Body weights of PRL1^−/−^/PRL2^+/−^ and PRL1^+/−^/PRL2^−/−^ males were also significantly less than those of PRL2^−/−^ males. **p < 0.01, ***p < 0.001 (Student’s t test). Data are representative of at least three independent experiments (mean and SD).

Given the lower expression of PRL1 relative to PRL2, loss of PRL1 may be compensated by the remaining PRL2, whereas loss of PRL2 cannot be fully rescued by PRL1^20^,^21^,^22^. However, since mice deficient in both PRL1 and PRL2 were embryonic lethal, we hypothesized that PRL1 and PRL2 have similar functions during development and that the combined total level of PRL1 and PRL2 dictates phenotype severity. To test this hypothesis, we then generated PRL1^+/−^/PRL2^−/−^ mice and PRL1^−/−^/PRL2^+/−^ mice. These mice also displayed a survival disadvantage as evidenced by the reduced birth rate (Table 2). PCR analysis was used to determine the deletion of PRL1 and PRL2 alleles (Fig. 1A). Deletion of the PRL protein was confirmed by Western blot on lung lysates from mice with different genotypes using PRL1/2 antibody (Fig. 1B). To examine the expression pattern of all PRLs, protein lysates from different organs were subjected to Western blot analysis using PRL1/2 and PRL3 antibodies. As shown in Figure S4A, both PRL1 and PRL2 are broadly expressed in all the organs we assayed. However, endogenous PRL3 is only moderately detectable in brain, lung, colon, small intestine, spleen, thymus, muscle and heart, which is consistent with the most recent report^9^. Given the same affinity and specificity of the PRL1/2 antibody towards PRL1 and PRL2, the intensity of the PRL1 band (upper band) and PRL2 band (lower band) reflects the relative level of endogenous PRL1 and PRL2. Overall, the level of PRL1 protein is lower than PRL2 in the organs we measured, from ~40% of PRL2 in testis to ~5% of PRL2 in muscle (Figure S4B). Supplemental Table 1 lists the combined PRL1 and PRL2 level relative to that of the wild-type in different organs of mice with different genotypes.

To characterize the overall phenotypic changes associated with PRL1/2 deletion, we first measured the body weight of more than twenty 4-weeks-old males and females with different genotypes. As mentioned above, PRL2^−/−^ mice exhibited a roughly 20% decrease in body weight compared with wild-type and PRL1^−/−^ mice. More strikingly, the body weights of PRL1^−/−^/PRL2^+/−^ and PRL1^+/−^/PRL2^−/−^ male mice decreased 38 ± 13% and 41 ± 10% in comparison with that of the wild-type males, and an additional 23 ± 16% and 27 ± 12% reduction was observed when compared to the body weight of PRL2^−/−^ males. Similarly, the body weights of PRL1^−/−^/PRL2^+/−^ and PRL1^+/−^/PRL2^−/−^ females were also decreased 30 ± 13% and 26 ± 14% compared to the wild-type controls (Fig. 1C,D). These observations revealed that the smaller body weight phenotype previously observed in PRL2^−/−^ mice was significantly intensified in PRL1^−/−^/PRL2^+/−^ and PRL1^+/−^/PRL2^−/−^ mice.

### Both PRL1 and PRL2 contribute to male reproductive ability

**Figure 2:**
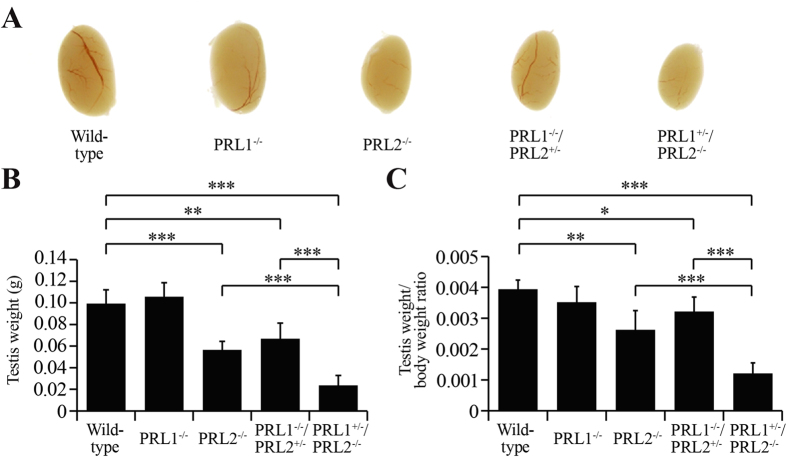
PRL1 also contributes to the normal testis development. (**A)** Representative testis picture from five different genotypes. (**B,C)** Testis weights (**B**) or testis weight/body weight ratios (**C**) from five different genotypes were determined. *p < 0.05, **p < 0.01, ***p < 0.001 (Student’s t test). Data are representative of at least three independent experiments (mean and SD).

One of the major phenotypes for PRL2^−/−^ mice is testicular atrophy^21^. Interestingly, both PRL1 and PRL2, but not PRL3, are expressed in testis, and the relative PRL1/PRL2 protein level is significantly higher in testis than those from other organs (Figure S4A). This finding suggests that PRL1 and PRL2, but not PRL3, may play essential roles in testis function. To determine whether both PRL1 and PRL2 are required for spermatogenesis, we focused on the testis phenotype in PRL1^−/−^/PRL2^+/−^ and PRL1^+/−^/PRL2^−/−^ mice. Anatomical examination was performed on adult males of different genotypes. Similar to our previous study^21^, the testis of PRL2 null mice is 43 ± 8% smaller than that of wild-type mice (Fig. 2A,B). Strikingly, deletion of a single allele of PRL1 gene from the PRL2-null mice caused an additional 59 ± 16% reduction of testis size, indicating that PRL1 is indeed involved in testis development (Fig. 2A,B). This is further strengthened by the observation that the testes of PRL1^−/−^/PRL2^+/−^ male mice were also markedly smaller than those of wild-type animals (33 ± 14% reduction) (Fig. 2A,B), despite the fact that testis development in either PRL1^−/−^ or PRL2^+/−^ mice is normal. Since the body weight of PRL1^−/−^/PRL2^+/−^ and especially PRL1^+/−^/PRL2^−/−^ male mice was also reduced (Fig. 1C), we normalized the testis weight to body weight and still found a significant reduction of the testis/body weight ratio in PRL1^−/−^/PRL2^+/−^ and especially PRL1^+/−^/PRL2^−/−^ mice. As shown in Fig. 2C, while the testis/body weight ratio observed in PRL2^−/−^ males decreased 33 ± 16% compared to the wild-type animals, deletion of one more PRL1 allele resulted in a 69 ± 9% reduction in the normalized testis size. Moreover, although no significant difference was observed in PRL1^−/−^ mice, the testis/body weight ratio of PRL1^−/−^/PRL2^+/−^ testis reduced 19 ± 12% compared to the wild-type mice (Fig. 2C).

**Figure 3:**
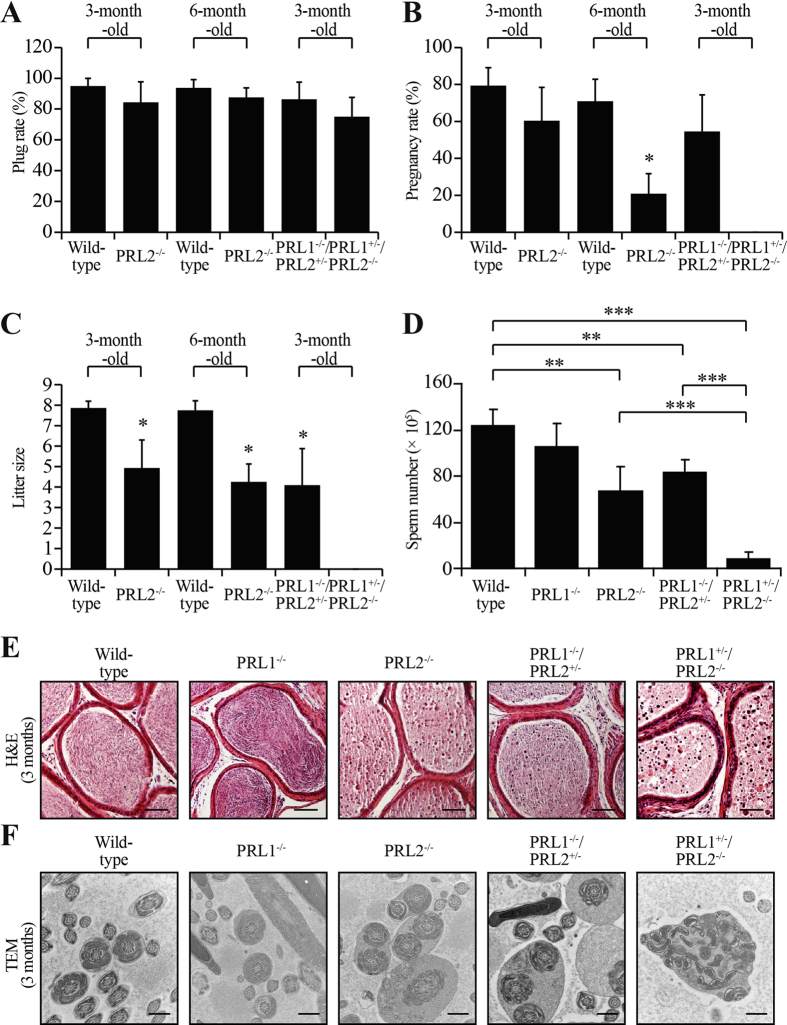
Deletion one PRL1 allele in PRL2^−/−^ results in male infertility due to no sperm production. (**A)** Sexual desire was measured by plug rate. (**B,C)** Reproductive performance was measured by pregnancy rate (**B**) and little size (**C**). (**D)** The sperm number was determined by calculating the sperms from epididymis. (**E)** Caudal epididymis sections from 3 months old male mice were histologically examined by H&E staining. Scale bar = 50 μm. (**F)** Electron microscopic analysis of epididymal sperm from the indicated genotypes. The typical “9 + 2” microtubule structure indicates the transverse section of the sperm. Scale bar = 500 nm. *p < 0.05, **p < 0.01, ***p < 0.001 (Student’s t test). Data are representative of at least three independent experiments (mean and SD).

To determine whether the reduced testis size affects male reproductivity, 3-months-old PRL1^−/−^/PRL2^+/−^ and PRL1^+/−^/PRL2^−/−^ male mice were mated with wild-type virgin females. These mice showed similar sexual desire as measured by the percentage of female mice plugged by the males (Fig. 3A). However, the percent pregnancy as well as the litter size from the PRL1^−/−^/PRL2^+/−^ mice group were significantly lower than those of the wild-type (Fig. 3B,C). More strikingly, none of the females that were plugged by PRL1^+/−^/PRL2^−/−^ male mice were pregnant, indicating that a single PRL1 allele deletion in PRL2 deficient mice results in male infertility (Fig. 3B,C). To further characterize the functionality of the testis, the number of spermatozoa from the epididymis of 3-months old male mice was counted using a hemocytometer. In agreement with previous findings^21^, the number of sperms from the PRL2^−/−^ mice was only 54 ± 18% of the wild-type controls (Fig. 3D). Similarly, the PRL1^−/−^/PRL2^+/−^ mice also had significantly decreased sperm counts (68 ± 9% of wild-type) (Fig. 3D), suggesting that both PRL1 and PRL2 contribute to sperm production. Not surprisingly, PRL1^+/−^/PRL2^−/−^ male mice lost 93 ± 5% of sperms in the epididymis (Fig. 3D), which explained their sterility. Consistent with this observation, H&E staining on caudal epididymis, where mature spermatozoa accumulate, failed to reveal any morphologically normal spermatozoa in the epididymis of PRL1^+/−^/PRL2^−/−^ mice (Fig. 3E). Although more round-shaped non-spermatozoa cells were also found in the epididymis of PRL2^−/−^ and PRL1^−/−^/PRL2^+/−^ mice, most of the cells were morphologically normal spermatozoa (Fig. 3E). To further characterize the structural defects of spermatozoa in the epididymis, we performed transmission electron microscopy (TEM) analysis. We found that sperms in PRL1 knockout mice displayed similar density and structural features as the sperms in the wild-type. In both PRL2^−/−^ and PRL1^−/−^/PRL2^+/−^ mice, some sperms were released as a cluster, indicating that these sperms failed to complete cell division. As expected, sperms were rarely observed in PRL1^+/−^/PRL2^−/−^ mice, and immature spermatocytes were released into epididymis, indicating increased germ cell apoptosis (Fig. 3F). Taken together, the data suggest that, in addition to PRL2, PRL1 also controls sperm production, which directly affects male reproductive ability. Given that the total levels of PRL1/2 in the testis of PRL1^−/−^, PRL2^−/−^, PRL1^−/−^/PRL2^+/−^ and PRL1^+/−^/PRL2^−/−^ mice are 70%, 30%, 35% and 15% of that of the wild-type, respectively (Supplemental Table 1), the data support our hypothesis that the reduction of the combined level of PRL1 and PRL2 correlates with the severity of testicular atrophy phenotype and the impairment of reproductive ability of male mice.

To further establish the functional redundancy between PRL1 and PRL2, we sought to demonstrate that the additional phenotypic defects caused by PRL1 deletion could be rescued by re-introduction of PRL2. To this end, we proceeded to generate a conditional PRL2 transgenic line (Supplemental Information). However, when we crossed the conditional PRL2 transgenic line to the whole body EIIA-Cre line (Figure S5A), we found that the PRL2 transgene was only expressed in the testis (Figure S5B). Although we do not understand why and how this happened, one possibility is that the increase levels of PRL2 in normal tissues may be intrinsically deleterious to normal cells, and consequently selected against in other tissues. Fortunately, this testis-specific PRL2 transgenic line (PRL2^Testis^) enabled us to test our hypothesis that the phenotype caused by PRL1 deletion could be rescued by re-expressing PRL2. To this end, we crossed the PRL2^Testis^ line with PRL2^−/−^, PRL1^−/−^/PRL2^+/−^ and PRL1^+/−^/PRL2^−/−^ mice. Western blot analysis indicated that the PRL2 transgene was expressed at ~50% of endogenous PRL2 in testis (Figure S5C). More importantly, the impaired reproductive ability and reduced testis weight phenotypes observed in PRL2^−/−^, PRL1^−/−^/PRL2^+/−^ and PRL1^+/−^/PRL2^−/−^ mice were rescued upon re-introduction of the PRL2 transgene (Figure S5D,E), suggesting that PRL1 deletion-induced defects in testis could indeed be rescued by re-expressing of PRL2.

### Total PRL1 and PRL2 level are important for maintaining spermatogenesis

**Figure 4:**
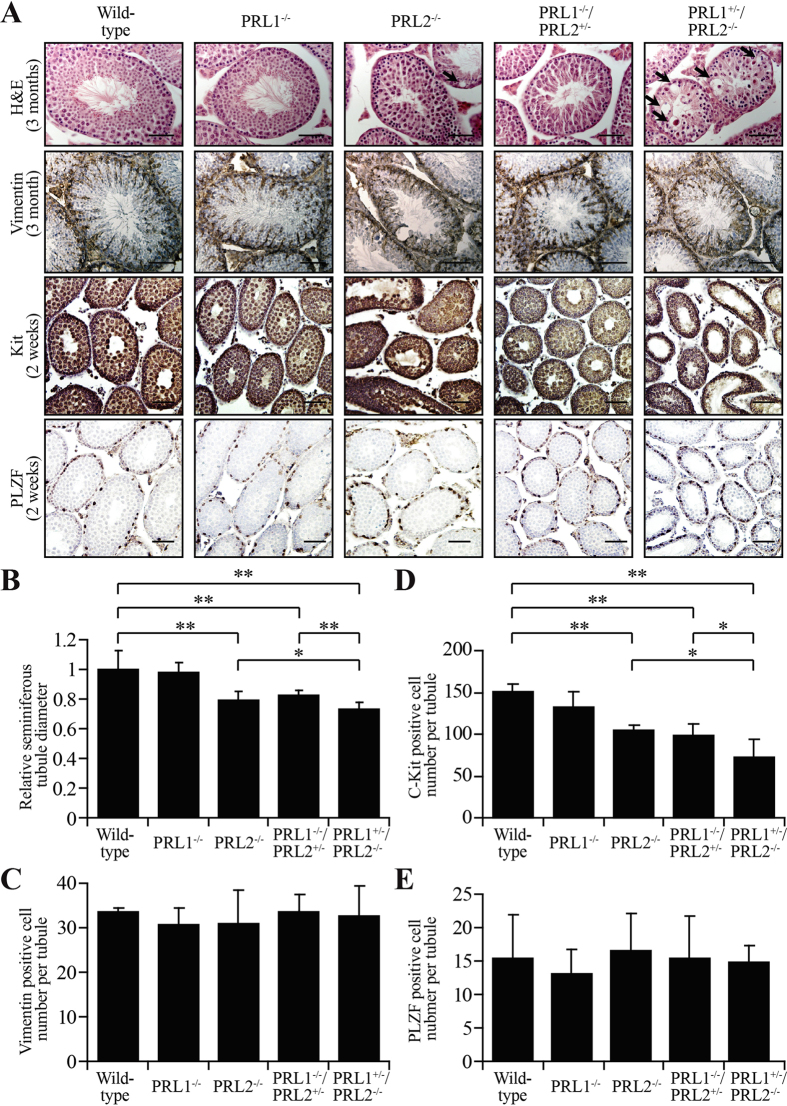
PRL1 also plays a role in maintaining spermatogenesis. (**A)** Testis sections from 3 months or 2 weeks old male mice were histologically examined by H&E staining and immunohistochemical staining for Vimentin, PLZF and Kit. (**B–E)** Relative seminiferous tubule diameters (**B**), number of Vimentin positive cell per seminiferous tubule (**C**), number of c-Kit positive cell per tubule (**D**) and number of PLZF positive cell per tubule (**E**) in testis sections from all five genotypes were quantificated. For each mouse, at least 20 tubules were counted. *p < 0.05, **p < 0.01 (Student’s t test). Data are representative of at least three independent experiments (mean and SD). Scale bar = 50 μm.

To further investigate how PRL1 is involved in controlling testis organ size and sperm production, the histological structures of 3-month-old male testis derived from different genotypes were analyzed by H&E staining. Even though the overall structures of the seminiferous tubules of 3-month-old male mice were similar between the different genotypes, the diameters of the seminiferous tubules were altered (Fig. 4A,B). Consistent with our previous study^21^, the diameter of the seminiferous tubules from the PRL2^−/−^ testis decreased 21 ± 6% in comparison to those from the wild-type and PRL1^−/−^ mice. There was also a 17 ± 3% and 27 ± 5% reduction in the diameter of the seminiferous tubules of the PRL1^−/−^/PRL2^+/−^ and PRL1^+/−^/PRL2^−/−^ testis, respectively (Fig. 4B). Moreover, loss of certain cell types was evidenced by the “empty space” observed inside of the seminiferous tubules of PRL2^−/−^ testis, and this was even more severe in the PRL1^+/−^/PRL2^−/−^ testis (Black arrows in Fig. 4A). In line with this observation, numerous round-shaped cells were released into the caudal epididymis of PRL2^−/−^ mice, and even more round-shaped cells were observed in the caudal epididymis of PRL1^+/−^/PRL2^−/−^ mice (Fig. 3E). In addition, re-expressing PRL2 in the testis successfully rescued the histological abnormality in PRL1^+/−^/PRL2^−/−^ testis (Figure S5F, top panel). These data indicated that the combined level of PRL1 and PRL2 is important for maintaining spermatogenesis and that the reduced testis size is mainly attributed to decreased cellularity in the seminiferous tubules.

To determine exactly which cell population is affected in the testis of different genotypes, we first examined the Sertoli cell number by Vimentin staining (previously used as a marker for Sertoli cell)^27^. As shown in Fig. 4A, the number of Sertoli cells from the 3-month-old male testis of the five genotypes was comparable (Fig. 4A,C), suggesting that the reduced cellularity is not due to loss of Sertoli cells. We then examined the germ cell number by PLZF staining (a specific marker for undifferentiated spermatogonia) and Kit staining (a specific marker for differentiating spermatogonia and primary spermatocytes)^28^,^29^. Our previous study of the PRL2^−/−^ mice suggested that differentiating spermatogonia and primary spermatocytes (Kit positive), but not undifferentiated spermatogonia (PLZF positive), were significantly reduced in PRL2^−/−^ testis at 2-week-old^21^. We speculated that deletion of one more PRL1 allele in PRL2^−/−^ testis will further aggravate the loss of Kit-positive cells. As shown in Fig. 4A,D,E, the number of PLZF-positive cells is comparable in all five genotypes, but the Kit-positive cell numbers varied between genotypes, suggesting that deletion of PRL1 and PRL2 primarily impact differentiating spermatogonia and primary spermatocytes. Consistent with our previous findings^21^, the number of Kit-positive cells in PRL2^−/−^ testis dropped 30 ± 4% of the wild-type controls (Fig. 4A,D). Comparable to the PRL2^−/−^ testis, the Kit-positive cell number also decreased 35 ± 10% in the PRL1^−/−^/PRL2^+/−^ testis (Fig. 4A,D). As expected, loss of Kit-positive cells was more pronounced in the PRL1^+/−^/PRL2^−/−^ testis (58 ± 8% less than the wild-type), in which many tubules are completely empty compared to the PRL2^−/−^ and PRL1^−/−^/PRL2^+/−^ testis (Fig. 4A,D). Taken together, the combination of PRL1^+/−^ and PRL2^−/−^ intensified the Kit-positive germ cell loss observed in PRL2^−/−^ testis, leading to a complete blockage of spermatogenesis and infertility.

### Sufficient level of total PRL1 and PRL2 are required to prevent germ cells from ectopic apoptosis

**Figure 5:**
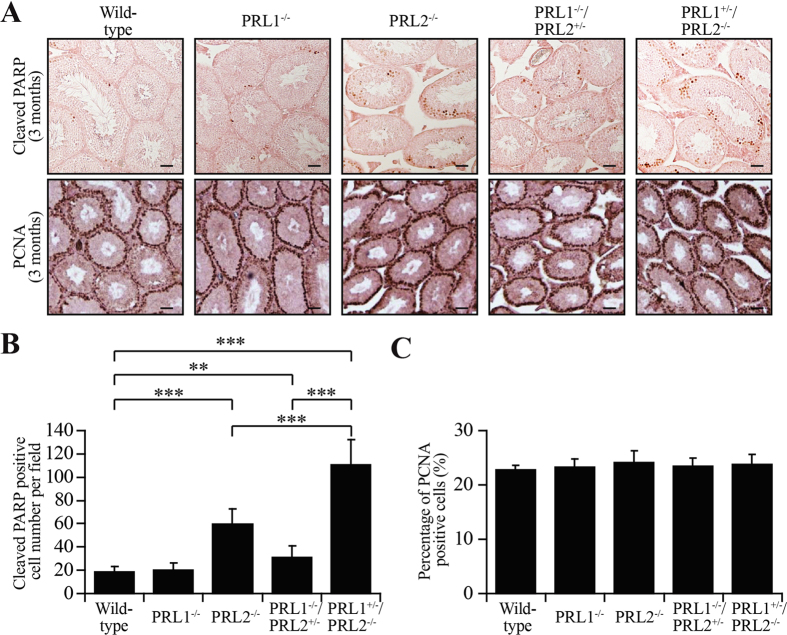
PRL1 is also required to prevent germ cells from apoptosis. (**A)** Testis sections from 3 month old male mice were histologically examined by immunohistochemical staining for Cleaved PARP and PCNA. (**B,C**) Number of cleaved-PARP positive cell per field (**B**) and percentage of PCNA positive cells per tubule (**C**) in testis sections from five genotypes were determined. For each mouse, at least 10 fields or 20 tubules were counted. **p < 0.01, ***p < 0.001 (Student’s t test). Data are representative of at least three independent experiments (mean and SD). Scale bar = 50 μm.

Spermatogenesis is a precisely controlled process including germ cell proliferation, differentiation, self-renewal and apoptosis. Germ cell loss occurs normally during spermatogenesis in all mammals, and control of germ cell apoptosis during spermatogenesis is especially important. To determine whether loss of Kit-positive germ cells was due to decreased proliferation or increased apoptosis, we performed immunohistochemistry with PCNA (Proliferating Cell Nuclear Antigen, a proliferation marker) staining and cleaved PARP (Poly ADP-Ribose Polymerase, an apoptosis marker) staining to label proliferating and apoptotic cells respectively. As shown in Fig. 5A,C, the percentage of proliferating cells in the testis was similar in all five genotypes. In contrast, the number of cleaved PARP positive cells was 3-fold higher in PRL2^−/−^ compared to wild-type or PRL1^−/−^ testis (Fig. 5A,B). PRL1^−/−^/PRL2^+/−^ mice also showed a 1.6 fold higher apoptosis compared to the control groups (Fig. 5A,B). More importantly, deletion of one PRL1 allele in PRL2^−/−^ mice led to an additional 3-fold increase in apoptosis in comparison to PRL2^−/−^ alone (Fig. 5A,B). The aggravated phenotype of PRL1^+/−^/PRL2^−/−^ mice compared to PRL2-null mice further supports an important role of PRL1 in spermatogenesis by promoting Kit-positive germ cell survival.

### Total PRL1 and PRL2 level negatively correlates with PTEN level

**Figure 6:**
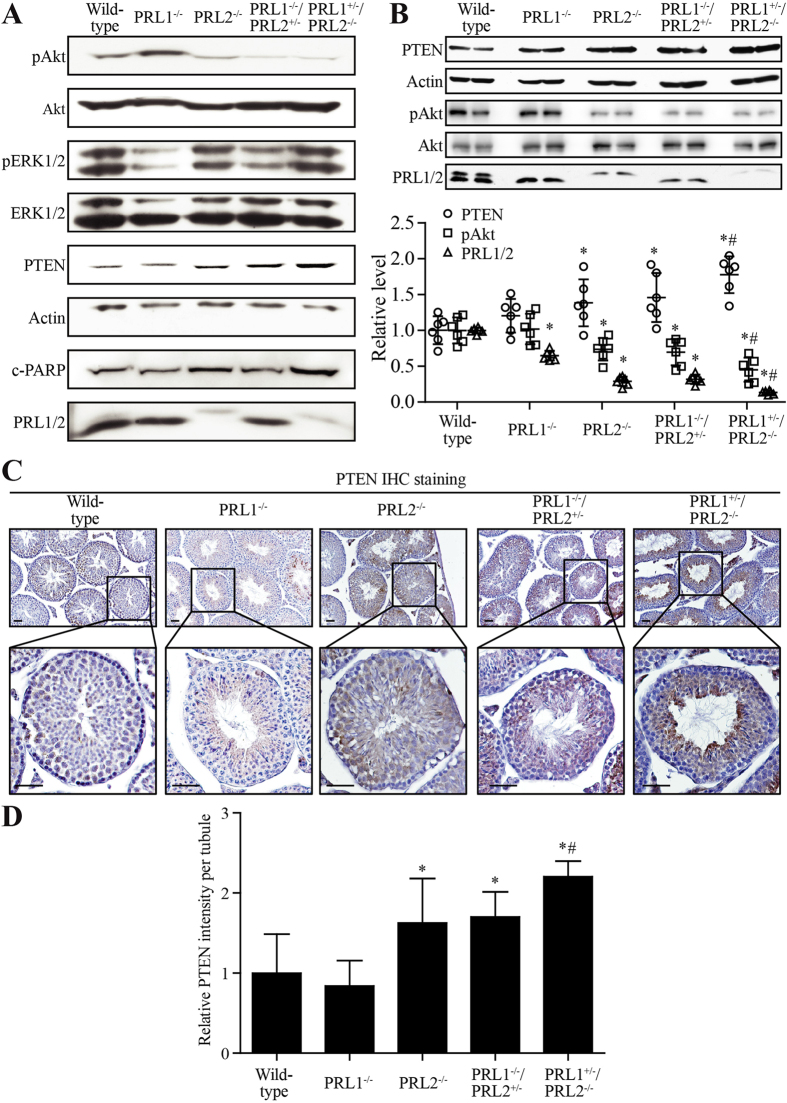
Total PRL1 and PRL2 negatively correlated with PTEN. (**A)** Alterations of key signaling pathway molecules in testis from different genotypes by Western blot. (**B)** Upper panel: germ cells isolated from testis of male mice with different genotypes were analyzed for PTEN, pAkt, Akt, Actin, PRL1 and PRL2 by Western blot. Lower panel: relative level of either PTEN/Actin, pAkt/Akt or total PRL1 and PRL2/Actin in isolated germ cells from different genotypes. (**C)** PTEN immunohistochemistry analysis in seminiferous tubules of testis with different genotypes. (**D**) Quantification of PTEN IHC staining by ImageJ IHC Image Analysis Toolbox. *p < 0.05 (Student’s t test) compared to wild-type; #p < 0.05 (Student’s t test) compared to PRL2^−/−^ and PRL1^−/−^/PRL2^+/−^. Scale bar = 50 μm.

PI3K/Akt is the major pathway that regulates the proliferation and survival of differentiating spermatogonia and spermatocytes^30^,^31^, and Akt1 knockout males exhibit impaired reproductive ability due to increased germ cell apoptosis^32^. Deletion of PRL2 leads to compromised Akt activation due to elevated PTEN^21^. To evaluate whether deletion of PRL1 also affects the PTEN/PI3K/Akt pathway, we next investigated the signaling pathways in whole testis lysate by Western blot. Similar to what we observed in PRL2^−/−^ mice, the testis from PRL1^−/−^/PRL2^+/−^ mice also showed increased PTEN level and reduced Akt activation (Fig. 6A). More strikingly, deletion of one PRL1 allele in PRL2^−/−^ mice further elevated the PTEN level and reduced pAkt, leading to more PARP cleavage (Fig. 6A). To further substantiate that the total PRL1 and PRL2 level negatively regulates PTEN expression in testis, we determined the total PRL1/2 and PTEN level as well as Akt activation in isolated germ cells by Western blot from all five genotypes (n = 6 for each genotype, Fig. 6B). Similar to the results from whole testis (Supplemental Table 1), the total PRL1/2 levels in germ cells isolated from PRL1^−/−^, PRL2^−/−^, PRL1^−/−^/PRL2^+/−^ and PRL1^+/−^/PRL2^−/−^ mice were 65 ± 7%, 29 ± 6%, 32 ± 6%, and 13 ± 2% of the wild-type controls (100 ± 4%). As expected, relative PTEN/Actin levels in germ cells isolated from PRL1^−/−^, PRL2^−/−^, PRL1^−/−^/PRL2^+/−^ and PRL1^+/−^/PRL2^−/−^ mice were 20 ± 23%, 39 ± 33%, 46 ± 34%, and 78 ± 26% higher than that of the wild-type (100 ± 19%). Consistently, the relative pAkt/Akt levels in germ cells isolated from PRL1^−/−^, PRL2^−/−^, PRL1^−/−^/PRL2^+/−^ and PRL1^+/−^/PRL2^−/−^ mice were 102 ± 21%, 73 ± 16%, 70 ± 19%, and 45 ± 17% of the wild-type (100 ± 18%). Consistently, immunohistochemistry analysis also revealed elevated PTEN in PRL2^−/−^, PRL1^−/−^/PRL2^+/−^ and, most strikingly, PRL1^+/−^/PRL2^−/−^ testes (Fig. 6C,D). Moreover, re-expression of the PRL2 transgene in PRL1^+/−^/PRL2^−/−^ testes successfully reduced PTEN level (Figure S5C,F, middle and bottom panels). Collectively, the data indicate that PRL1, like PRL2, is also involved in PTEN downregulation and there is a negative correlation between the total PRL1/2 level and the PTEN level.

Given the significant role of PTEN in down-regulating PI3K/Akt signaling pathway, it is understandable that loss of heterozygosity in PTEN^+/−^ mice causes spontaneous tumorigenesis including testicular germ cell cancer^33^. PTEN is one of the most well-known tumor suppressors that are frequently mutated, and commonly down-regulated in cancer^34^. Loss of function PTEN mutations or even a 20% reduction in PTEN expression can amplify PI3K signaling and promote tumorigenesis in a variety of experimental models of cancer^35^. It has been previously reported that loss of PTEN leads to testicular teratoma^36^. To further evaluate the clinical relevance of our observed negative correlation between PTEN and PRL1 and PRL2, we analyzed PRL1, PRL2 and PTEN mRNA level in normal and testicular cancer patient samples from a publicly available microarray dataset using the Affymetrix U133A platform^37^. We found that both PRL1 and PRL2 level are significantly elevated, while PTEN level is significantly decreased in testicular cancer patients (n = 184) compared to normal testis samples (n = 13) (Figure S6). Taken together, our finding that both PRL1 and PRL2 are involved in the negative regulation of PTEN offers a plausible explanation for the positive roles played by PRL1 and PRL2 in promoting cell proliferation and survival as well as tumorigenesis. Together with the previous finding that PRL3 also down-regulates PTEN through increasing its degradation^17^, our studies also suggest that the oncogenic potential of all PRLs is underscored by their ability to down-regulate PTEN thereby activating the Akt pathway.

In summary, our study suggests that the lack of significant phenotypic effect of PRL1 deletion could be explained, in part, by an insufficient decrease in the combined level of PRL1 and PRL2, due to the relatively lower PRL1 versus PRL2 expression. Our study also elucidates a previously undescribed cooperative function of both PRL1 and PRL2 in spermatogenesis by regulating PTEN/Akt signaling pathway. Our results not only suggest an essential role of both PRL1 and PRL2 in testicular development by maintaining spermatogonia stem cell survival, but also indicate a potential disease mechanism in which overexpression of PRL1 and PRL2 results in reduced PTEN level in testis, thereby leading to testicular cancer. From a clinical point of view, PRL1 and PRL2 may be potential biomarkers and therapeutic targets for testicular cancers and drug targets for male contraception.

## Material and Methods

### Animals

Generation of PRL2 deficient mice was described previously using “gene-trapping” method^20^. Heterozygous gene trap embryonic stem (ES) cell line (Cell No.: CC0606; Mouse strain: 129P2/OlaHsd) containing an insertional mutation in mouse PRL1 locus was purchased from Mutant Mouse Regional Resource Centers (MMRRC). The ES cells were injected into blastocysts from C57BL/6J mice in the Transgenic and Knock-Out Mouse Core at Indiana University Simon Cancer Center, and the resulting chimeric males with successful germ line transmission of the mutant allele were intercrossed with C57BL/6J females to generate F1 offspring. Genotyping of F1 offspring was performed by PCR. Genotyping primers used for detecting wild-type alleles are GCAGACAAGTGAACTGTAGAAATTC and AGTGTAGCACACTTCTACCGTTCCA, and for detecting mutant alleles are GCAGACAAGTGAACTGTAGAAATTC and ACGACGGGATCCTCTAGAG. PRL1^+/−^/PRL2^+/−^ intercrossing was used to generate PRL1^−/−^/PRL2^+/−^ and PRL1^+/−^/PRL2^−/−^ mice. Mice used in this study were all on a C57BL6/129P2 mixed genetic background. All experiments on mice were carried out in accordance with the Guide for Care and Use of Laboratory Animals of the National Institutes of Health, and animal protocols were approved by the Institutional Animal Care and Use Committees of Indiana University (Protocol Number 10504).

### RNA extraction and quantitative RT-PCR

Total RNA was extracted from tissues using Trizol reagent (Invitrogen) and treated with DNase I (Promega). Reverse transcription was performed using the SuperScript III one-step RT-PCR system (Invitrogen) at 55 °C with gene-specific primers. Quantitative PCR was performed using SYBR Green I master mix on a LightCycler 480 (Roche Applied Science), with RPL7 as the housekeeping gene control. RT-PCR primers are GAAGAGGCCGTCTCCACTAA and ATCCCAGGATTGCGATTACA for detecting PRL1, GGCTGTAACAGGGTGGAAGA and GCCACCAACATCTGGGTACT for detecting PRL2, and GCTGCGGATTGTGGAGCCATAC and CCTCCATGCAGATGATGCCAAAC for detecting RPL7.

### Western Blot analysis

Tissues were lysed in lysis buffer supplied with phosphatase and protease inhibitor mixture (Roche Applied Science). Equal amounts of protein were resolved by SDS-PAGE, transferred to nitrocellulose membrane and subjected to immunoblotting. Anti- PRL1, anti-PRL1/2 and anti-PRL3 antibodies were described in ref. 38. All other antibodies were from Cell Signaling Technology.

### Reproductive performance evaluation

Age matched wild-type, PRL1^−/−^, PRL2^−/−^, PRL1^−/−^/PRL2^+/−^ and PRL1^+/−^/PRL2^−/−^ males were each mated with 4 wild-type virgin females every day for 6 consecutive days. Vaginal plugs were monitored every morning, and plugged females were removed and replaced with virgin females. After 6 days, the total number of females plugged by each male was counted. Plugged females were kept for an additional month to determine the number of pregnant mice and their litter sizes. The percentage of pregnant mice among the plugged and the average litter size were calculated for each male.

### Histology

Tissues were fixed in 4% paraformaldehyde (PFA) overnight at 4 °C, embedded in paraffin, serially sectioned (7 μm), and stained with H&E according to standard methods. For immunohistochemistry, de-paraffined and hydrated sections were subjected to antigen retrieval by boiling in 10 mM sodium citrate for 20 min. Sections were then incubated with diluted antibodies (1:50–1:400) at 4 °C overnight. Signals were detected by VECTASTAIN Elite ABC kit and developed using DAB substrate from Vector laboratory (Burlingame, CA). Antibodies used were PLZF, Kit (Santa Cruz Biotechnology, CA), Vimentin, PCNA, cleaved PARP and PTEN (Cell Signaling Technology, MA). Images were captured on a Leica DM2500 stereomicroscope. All images are representative of at least 3 samples.

### Testicular cell isolation

Testes isolated from wild-type, PRL1^−/−^, PRL2^−/−^, PRL1^−/−^/PRL2^+/−^ and PRL1^+/−^/PRL2^−/−^ males were de-capsulated and digested in DMEM containing 1 mg/mL collagenase I at 32 °C for 20 min with gentle agitation. Released interstitial cells were removed, and seminiferous tubules were washed twice with DMEM. Seminiferous tubules were then subjected to second enzymatic digestion in DMEM with 1 mg/mL collagenase I, 0.5 mg/mL trypsin, 50 U/mL hyaluronidase, and 100 μg/mL DNase I at 32 °C for 30 min with gentle agitation. Seminiferous tubules were pipetted up and down for 10 times to disassociate the cells. The cell clumps were removed by passing through a 70 μm nylon filter, and the single cell preparation was incubated in a culture dish in DMEM at 32 °C with 5% CO_2_ for 3 h to allow Sertoli cells and peritubular cells to attach. Germ cells in the suspension were then counted and used immediately.

### Sperm count

Caudal epididymis were isolated from age-matched wild-type, PRL1^−/−^, PRL2^−/−^, PRL1^−/−^/PRL2^+/−^ and PRL1^+/−^/PRL2^−/−^ males, minced in 10 mL BWW buffer (NaCl 5.54 g/L, KCl 0.356 g/L, CaCl_2_•2H_2_O 0.250 g/L, KH_2_PO_4_ 0.162 g/L, MgSO_4_•7H_2_O 0.294 g/L, NaHCO_3_ 2.1 g/L, glucose 1.0 g/L, Sodium pyruvic acid 0.03 g/L, BSA 3.5 g/L), and incubated at 32 °C for 15 min. After mixed by pipetting, the motile and total sperm numbers were counted using hemocytometer.

### Statistical analysis

Statistical significant differences were calculated using student’s t-test or χ^2^ test and represented by asterisks: *p < 0.05, **p < 0.01, ***p < 0.001.

## Additional Information

**How to cite this article**: Bai, Y. *et al.* Role of phosphatase of regenerating liver 1 (PRL1) in spermatogenesis. *Sci. Rep.* **6**, 34211; doi: 10.1038/srep34211 (2016).

## Supplementary Material

Supplementary Information

## Figures and Tables

**Table 1:** Distribution of offspring by PRL1 or PRL2 heterozygous-heterozygous mating at weaning indicates a survival disadvantage of PRL1 and PRL2 knockout mice.

	+/+	+/−	−/−	n	χ^2^	*p*
**PRL1**						
Observed	187	353	143	683	6.44	<0.05
Expected	171	342	171			
**PRL2**						
Observed	139	307	73	519	34.18	<0.001
Expected	130	260	130			

**Table 2:** Distribution of offspring by double heterozygous-double heterozygous (PRL1^+/−^/PRL2^+/−^) mating at weaning indicates embryonic lethality of PRL1^−/−^/PRL2^−/−^ double knockout mice and a survival disadvantage of PRL1^−/−^/PRL2^+/−^ and PRL1^+/−^/PRL2^−/−^ mice.

PRL1	+/+			+/−			−/−			n	χ^2^	*p*
PRL2	+/+	+/−	−/−	+/+	+/−	−/−	+/+	+/−	−/−			
Observed	22	56	30	54	110	27	18	35	0	352	45.10	<0.001
Expected	22	44	22	44	88	44	22	44	22			

## References

[b1] Diamond R. H., Cressman D. E., Laz T. M., Abrams C. S. & Taub R. PRL-1, a unique nuclear protein tyrosine phosphatase, affects cell growth. Mol Cell Biol. 14, 3752–3762 (1994). 8196618 10.1128/mcb.14.6.3752-3762.1994 PMC358742

[b2] Cates C. A. *et al.* Prenylation of oncogenic human PTP(CAAX) protein tyrosine phosphatases. Cancer Lett. 110, 49–55 (1996). 9018080 10.1016/s0304-3835(96)04459-x

[b3] Zeng Q. *et al.* PRL-3 and PRL-1 promote cell migration, invasion, and metastasis. Cancer Res. 63, 2716–2722 (2003). 12782572

[b4] Zeng Q., Hong W. & Tan Y. H. Mouse PRL-2 and PRL-3, two potentially prenylated protein tyrosine phosphatases homologous to PRL-1. Biochem Biophys Res Commun. 244, 421–427, doi: 10.1006/bbrc.1998.8291 (1998). 9514946

[b5] Bessette D. C., Qiu D. & Pallen C. J. PRL PTPs: mediators and markers of cancer progression. Cancer Metast Rev. 27, 231–252, doi: 10.1007/s10555-008-9121-3 (2008). 18224294

[b6] Matter W. F. *et al.* Role of PRL-3, a human muscle-specific tyrosine phosphatase, in angiotensin-II signaling. Biochem Biophys Res Commun. 283, 1061–1068, doi: 10.1006/bbrc.2001.4881 (2001). 11355880

[b7] Dumaual C. M., Sandusky G. E., Crowell P. L. & Randall S. K. Cellular localization of PRL-1 and PRL-2 gene expression in normal adult human tissues. J Histochem Cytochem. 54, 1401–1412, doi: 10.1369/jhc.6A7019.2006 (2006). 16957164 PMC3958126

[b8] Gungabeesoon J., Tremblay M. L. & Uetani N. Localizing PRL-2 expression and determining the effects of dietary Mg on expression levels. Histochem Cell Biol. doi: 10.1007/s00418-016-1427-6 (2016). 27015884

[b9] Zimmerman M. W., Homanics G. E. & Lazo J. S. Targeted deletion of the metastasis-associated phosphatase Ptp4a3 (PRL-3) suppresses murine colon cancer. Plos one 8, e58300, doi: 10.1371/journal.pone.0058300 (2013). 23555575 PMC3610886

[b10] Stephens B. J., Han H., Gokhale V. & Von Hoff D. D. PRL phosphatases as potential molecular targets in cancer. Mol Cancer Ther. 4, 1653–1661, doi: 10.1158/1535-7163.MCT-05-0248 (2005). 16275986

[b11] Wang J., Kirby C. E. & Herbst R. The tyrosine phosphatase PRL-1 localizes to the endoplasmic reticulum and the mitotic spindle and is required for normal mitosis.J Biol Chem. 277, 46659–46668, doi: 10.1074/jbc.M206407200 (2002). 12235145

[b12] Wang Q., Holmes D. I., Powell S. M., Lu Q. L. & Waxman J. Analysis of stromal-epithelial interactions in prostate cancer identifies PTPCAAX2 as a potential oncogene. Cancer Lett. 175, 63–69 (2002). 11734337 10.1016/s0304-3835(01)00703-0

[b13] Werner S. R. *et al.* Enhanced cell cycle progression and down regulation of p21(Cip1/Waf1) by PRL tyrosine phosphatases. Cancer Lett. 202, 201–211 (2003). 14643450 10.1016/s0304-3835(03)00517-2

[b14] Liang F. *et al.* PRL3 promotes cell invasion and proliferation by down-regulation of Csk leading to Src activation. J Biol Chem. 282, 5413–5419, doi: 10.1074/jbc.M608940200 (2007). 17192274

[b15] Fiordalisi J. J., Keller P. J. & Cox A. D. PRL tyrosine phosphatases regulate rho family GTPases to promote invasion and motility. Cancer Res. 66, 3153–3161, doi: 10.1158/0008-5472.CAN-05-3116 (2006). 16540666

[b16] Achiwa H. & Lazo J. S. PRL-1 tyrosine phosphatase regulates c-Src levels, adherence, and invasion in human lung cancer cells. Cancer Res. 67, 643–650, doi: 10.1158/0008-5472.CAN-06-2436 (2007). 17234774

[b17] Wang H. *et al.* PRL-3 down-regulates PTEN expression and signals through PI3K to promote epithelial-mesenchymal transition. Cancer Res. 67, 2922–2926, doi: 10.1158/0008-5472.CAN-06-3598 (2007). 17409395

[b18] Bai Y. *et al.* PRL-1 protein promotes ERK1/2 and RhoA protein activation through a non-canonical interaction with the Src homology 3 domain of p115 Rho GTPase-activating protein. J Biol Chem. 286, 42316–42324, doi: 10.1074/jbc.M111.286302 (2011). 22009749 PMC3234939

[b19] Saha S. *et al.* A phosphatase associated with metastasis of colorectal cancer. Science 294, 1343–1346, doi: 10.1126/science.1065817 (2001). 11598267

[b20] Dong Y. *et al.* Phosphatase of regenerating liver 2 (PRL2) is essential for placental development by down-regulating PTEN (Phosphatase and Tensin Homologue Deleted on Chromosome 10) and activating Akt protein. J Biol Chem. 287, 32172–32179, doi: 10.1074/jbc.M112.393462 (2012). 22791713 PMC3442547

[b21] Dong Y. *et al.* Phosphatase of regenerating liver 2 (PRL2) deficiency impairs Kit signaling and spermatogenesis. J Biol Chem. 289, 3799–3810, doi: 10.1074/jbc.M113.512079 (2014). 24371141 PMC3916576

[b22] Kobayashi M. *et al.* PRL2/PTP4A2 Phosphatase Is Important for Hematopoietic Stem Cell Self-Renewal. Stem Cells 32, 1956–1967, doi: 10.1002/Stem.1672 (2014). 24753135 PMC4063874

[b23] Hardy S. *et al.* The protein tyrosine phosphatase PRL-2 interacts with the magnesium transporter CNNM3 to promote oncogenesis. Oncogene 34, 986–995, doi: 10.1038/onc.2014.33 (2015). 24632616

[b24] Yamazaki D., Funato Y., Miyata H., Ikawa M. & Miki H. Complementary role of CNNM2 in sperm motility and Ca(2+) influx during capacitation. Biochem Biophys Res Commun. 474, 441–446, doi: 10.1016/j.bbrc.2016.05.001 (2016). 27150626

[b25] Yamazaki D. *et al.* The Mg2+ transporter CNNM4 regulates sperm Ca2+ homeostasis and is essential for reproduction. J Cell Sci. 129, 1940–1949, doi: 10.1242/jcs.182220 (2016). 27006114

[b26] Zimmerman M. W. *et al.* Protein-tyrosine phosphatase 4A3 (PTP4A3) promotes vascular endothelial growth factor signaling and enables endothelial cell motility. J Biol Chem. 289, 5904–5913, doi: 10.1074/jbc.M113.480038 (2014). 24403062 PMC3937659

[b27] Paranko J., Kallajoki M., Pelliniemi L. J., Lehto V. P. & Virtanen I. Transient coexpression of cytokeratin and vimentin in differentiating rat Sertoli cells. Dev Biol. 117, 35–44 (1986). 2427374 10.1016/0012-1606(86)90345-3

[b28] Buaas F. W. *et al.* Plzf is required in adult male germ cells for stem cell self-renewal. Nat Genet 36, 647–652 (2004). 15156142 10.1038/ng1366

[b29] Vincent S. *et al.* Stage-specific expression of the Kit receptor and its ligand (KL) during male gametogenesis in the mouse: a Kit-KL interaction critical for meiosis. Development 125, 4585–4593 (1998). 9778516 10.1242/dev.125.22.4585

[b30] Lee J. *et al.* Akt mediates self-renewal division of mouse spermatogonial stem cells. Development 134, 1853–1859, doi: 10.1242/dev.003004 (2007). 17428826

[b31] Ciraolo E. *et al.* Essential role of the p110beta subunit of phosphoinositide 3-OH kinase in male fertility. Mol Biol Cell 21, 704–711, doi: 10.1091/mbc.E09-08-0744 (2010). 20053680 PMC2828958

[b32] Chen W. S. *et al.* Growth retardation and increased apoptosis in mice with homozygous disruption of the Akt1 gene.Gene Dev. 15, 2203–2208, doi: 10.1101/gad.913901 (2001). 11544177 PMC312770

[b33] Suzuki A. *et al.* High cancer susceptibility and embryonic lethality associated with mutation of the PTEN tumor suppressor gene in mice. Curr Biol. 8, 1169–1178 (1998). 9799734 10.1016/s0960-9822(07)00488-5

[b34] Salmena L., Carracedo A. & Pandolfi P. P. Tenets of PTEN tumor suppression. Cell 133, 403–414 (2008). 18455982 10.1016/j.cell.2008.04.013

[b35] Alimonti A. *et al.* Subtle variations in Pten dose determine cancer susceptibility. Nat Genet 42, 454–458 (2010). 20400965 10.1038/ng.556 PMC3118559

[b36] Kimura T. *et al.* Conditional loss of PTEN leads to testicular teratoma and enhances embryonic germ cell production. Development 130, 1691–1700 (2003). 12620992 10.1242/dev.00392

[b37] Shin G. *et al.* GENT: gene expression database of normal and tumor tissues. Cancer Inform. 10, 149–157, doi: 10.4137/CIN.S7226 (2011). 21695066 PMC3118449

[b38] Li J. *et al.* Generation of PRL-3- and PRL-1-specific monoclonal antibodies as potential diagnostic markers for cancer metastases. Clin Cancer Res. 11, 2195–2204, doi: 10.1158/1078-0432.CCR-04-1984 (2005). 15788667

