# Supplementary material for: Role of phosphatase of regenerating liver 1 (PRL1) in spermatogenesis
Source: Sci Rep. 2016 Sep 26;6:34211. doi: 10.1038/srep34211 (PMC5035919; doi:10.1038/srep34211)
Supplement: Supplementary Information [file srep34211-s1.pdf]

Supplemental Information

Role of phosphatase of regenerating liver 1 (PRL1) in spermatogenesis

Yunpeng Bai[∥], Lujuan Zhang[#], Hongming Zhou[#], Yuanshu Dong[#‡], Qi Zeng[§], Weinian Shou[†], and Zhong-Yin Zhang[∥#]*

[∥]Departments of Medicinal Chemistry and Molecular Pharmacology and of Chemistry, Purdue Center for Cancer Research, and Purdue Center for Drug Discovery, Purdue University, 575 Stadium Mall Drive, West Lafayette, IN 47907, USA, [#]Department of Biochemistry and Molecular Biology, and [†]Department of Pediatrics, Indiana University School of Medicine, Indianapolis, IN 46202, USA, and [§]Institute of Molecular and Cell Biology, A*STAR (Agency for Science, Technology and Research), 61 Biopolis Drive, Proteos, Singapore 138673, Republic of Singapore.

*To whom correspondence should be addressed: zhang-zy@purdue.edu.

[‡]Current address: Institute of biological and medical sciences, Soochow University, Suzhou, Jiangsu, China.

GENERATION OF PRL2 TRANSGENIC MICE

Plasmid construction for transgenic vector piZEG-PRL2. The transgenic vector piZEG-PRL2 was constructed based on the piZEG plasmid (Novak et al., 2000). piZEG contains a CAG (chicken beta-actin) promoter, which is followed by a loxP-flanked sequence containing LacZ, a neomycin selection cassette, and a transcriptional STOP sequence. The CAG promoter also drives the expression of enhanced green fluorescent protein (EGFP) coding regions linked by an internal ribosomal entry site (IRES). The piZEG plasmid was purchased from the Miami Mice Research Center (http://www.cancerbiology.org/index.html). To generate the piZEG-PRL2 expression vector, the ~500bp PRL2 coding region, flanked by BglII and XhoI restriction enzyme sites, was amplified from the mouse muscle cDNA and inserted into the BglII-XhoI sites of piZEG. The piZEG-PRL2 construct was purified and used for microinjection.

Generation of iZEG-PRL2 transgenic mice. The microinjection was performed in the Transgenic and Knock-Out Mouse Core at Indiana University Simon Cancer Center. The derived mice were screened by PCR using common primers iZEG_F1 (5'-TCGATGCAGGATAACTTCGTAT-3') and iZEG_R2 (5'-GATAAGCTTGATATCGAATTCC-3') that flank the PRL2 cDNA (584 bp fragment). With the PCR, three animals (#8, #11 and #66) from 90 pups were identified to be positive for the transgene insertion. In order to examine whether the promoter is still active following insertion, the tail tips from the identified three animals were processed for examining LacZ expression, which is directly under control of the CAG promoter. Only #8 and #11 were positive for LacZ expression and were further analyzed for germ-line transmission of transgene by crossing to wild-type. F1 pups from founder #11 have revealed successful germ-line transmission.

Generation of EIIA-Cre/iZEG-PRL2 double transgenic mice. iZEG-PRL2 transgenic mice were mated with EIIA-Cre transgenic mice to generate EIIA-Cre/iZEG-

PRL2 double transgenic offsprings, which were genotyped via PCR using primers Cre_F (5'-TGCCAGGATCAGGGTTAAAG-3') and Cre_R (5'-TGCATGATCTCCGGTATTGA-3') to detect Cre (402 bp fragment) and EGFR_F (5'-ACGTAAACGGCCACAAGTTC-3') and EGFR_R (5'-CTGGGTGCTCAGGTAGTGGT-3') to detect EGFP (551 bp fragment). Cre-mediated recombination was detected by PCR analysis using the primers iZEG_F4 (5'-CTGGTTATTGTGCTGTCTCATCA-3') and iZEG_R1 (5'-GGCTTCGGCCAGTAACGTTAG-3'), which will produce a 688 bp fragment.

Examination of PRL2 transgene expression in EIIA-Cre/iZEG-PRL2 double transgenic mice. Since the EIIA promoter is supposed to be ubiquitously active, to examine the PRL2 transgene expression in the transgenic mice, we isolated different organs from the transgenic mice and measured the PRL2 expression by using EGFP antibody (an indicator of PRL2 transgene expression). Unfortunately, our PRL2 transgenic line only has PRL2 overexpression in testis, but not other organs (Supplemental Figure 6B). Therefore, we have generated a testis-specific PRL2 transgenic line ($PRL2^{Testis}$).

Generation of $PRL2^{-/-}/PRL2^{Testis}$ mice, $PRL1^{-/-}/PRL2^{+/-}/PRL2^{Testis}$ mice and $PRL1^{+/-}/PRL2^{-/-}/PRL2^{Testis}$ mice. In order to detect if PRL2 overexpression in testis could rescue the testis phenotypes observed in $PRL2^{-/-}$/, $PRL1^{-/-}/PRL2^{+/-}$ and $PRL1^{+/-}/PRL2^{-/-}$ mice, $PRL2^{Testis}$ mice were crossed with $PRL1^{+/-}/PRL2^{+/-}$ mice to generate $PRL1^{+/-}/PRL2^{+/-}/PRL2^{Testis}$ mice. Then the $PRL1^{+/-}/PRL2^{+/-}/PRL2^{Testis}$ mice were crossed with $PRL1^{+/-}/PRL2^{+/-}$ mice to generate $PRL2^{-/-}/PRL2^{Testis}$, $PRL1^{-/-}/PRL2^{+/-}/PRL2^{Testis}$ *and* $PRL1^{+/-}/PRL2^{-/-}/PRL2^{Testis}$ mice.

Novak, A., Guo, C., Yang, W., Nagy, A. and Lobe, C. G. (2000). Z/EG, a double reporter mouse line that expresses enhanced green fluorescent protein upon Cre-mediated excision. *Genesis* **28**, 147-155.

Supplemental Figure 1

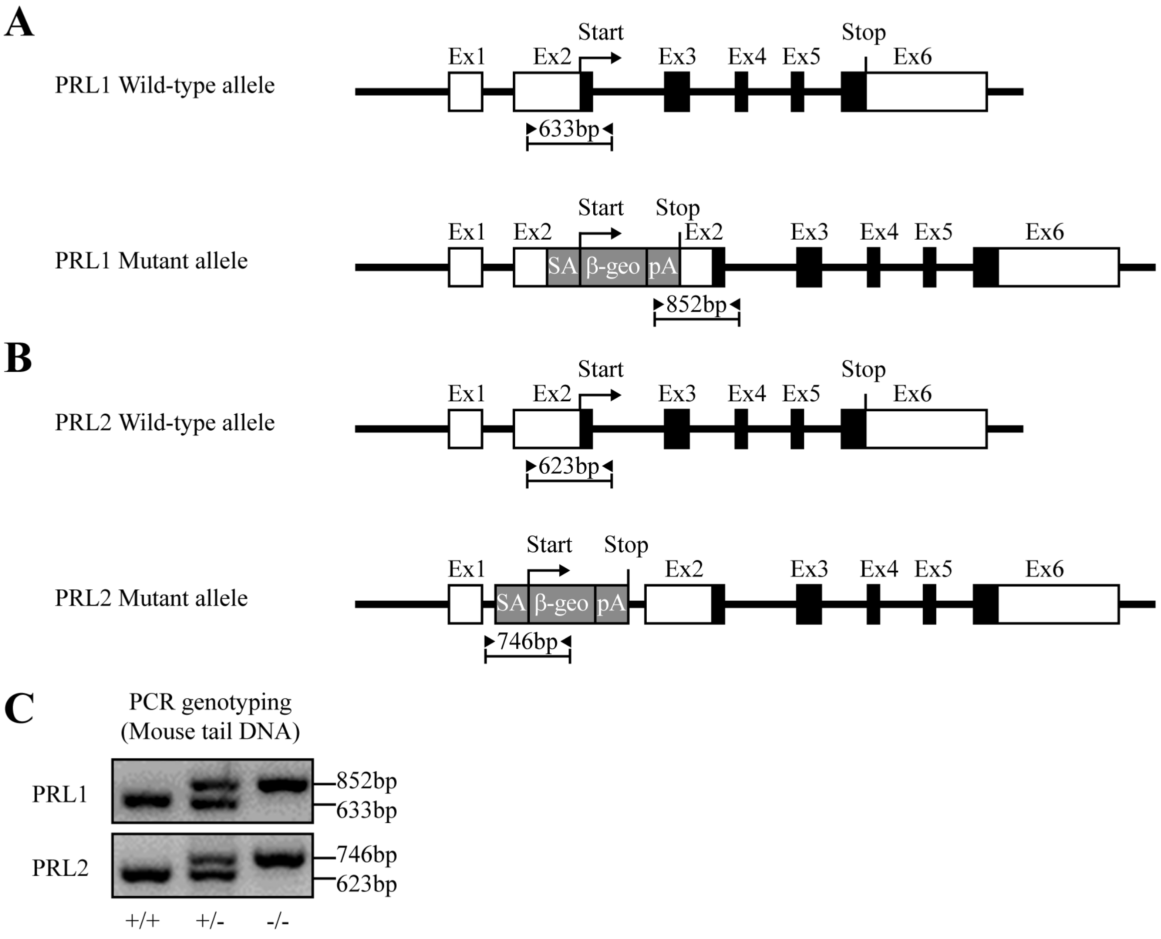

Supplemental Figure 1. Creation of $PRL1^{-/-}$ and $PRL2^{-/-}$ mice. A. Diagram showing the PRL1 wild-type allele and PRL1 mutant allele. B. Diagram showing the PRL2 wild-type allele and PRL2 mutant allele. C. Genotyping to identify PRL1 and PRL2 wild-type allele and PRL1 and PRL2 mutant allele.

Supplemental Figure 2

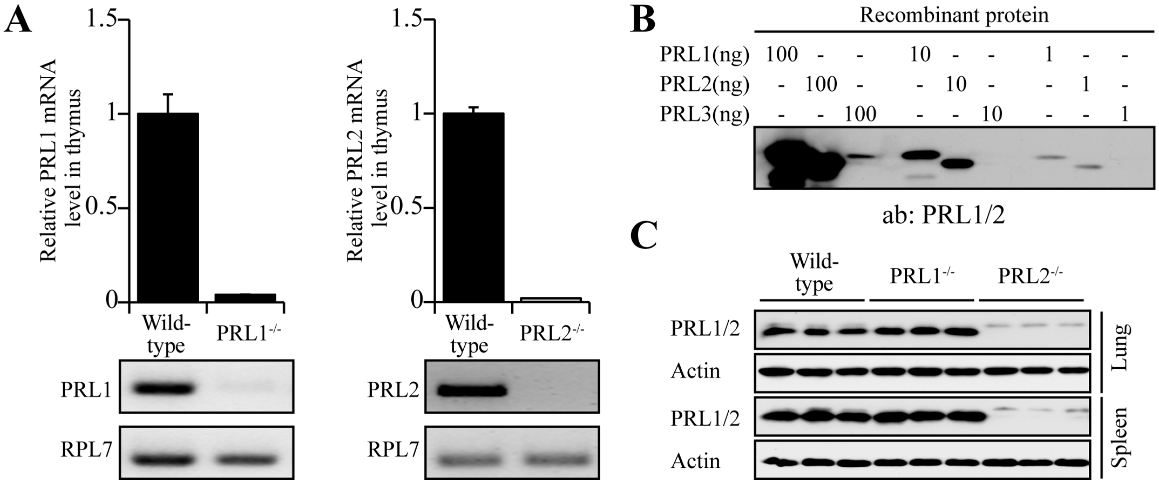

Supplemental Figure 2. Validation of PRL1 and PRL2 deletion. A. RT-PCR confirms PRL1 and PRL2 deletion in thymus. B. PRL1/2 antibody recognizes both PRL1 and PRL2 at the similar level by measuring 100 ng, 10 ng and 1 ng of recombinant PRL1, PRL2 and PRL3 by Western blot using PRL1/2 antibody. C. Western blot confirms PRL1 and PRL2 deletion in lung and spleen by Western blot using PRL1/2 antibody.

Supplemental Figure 3

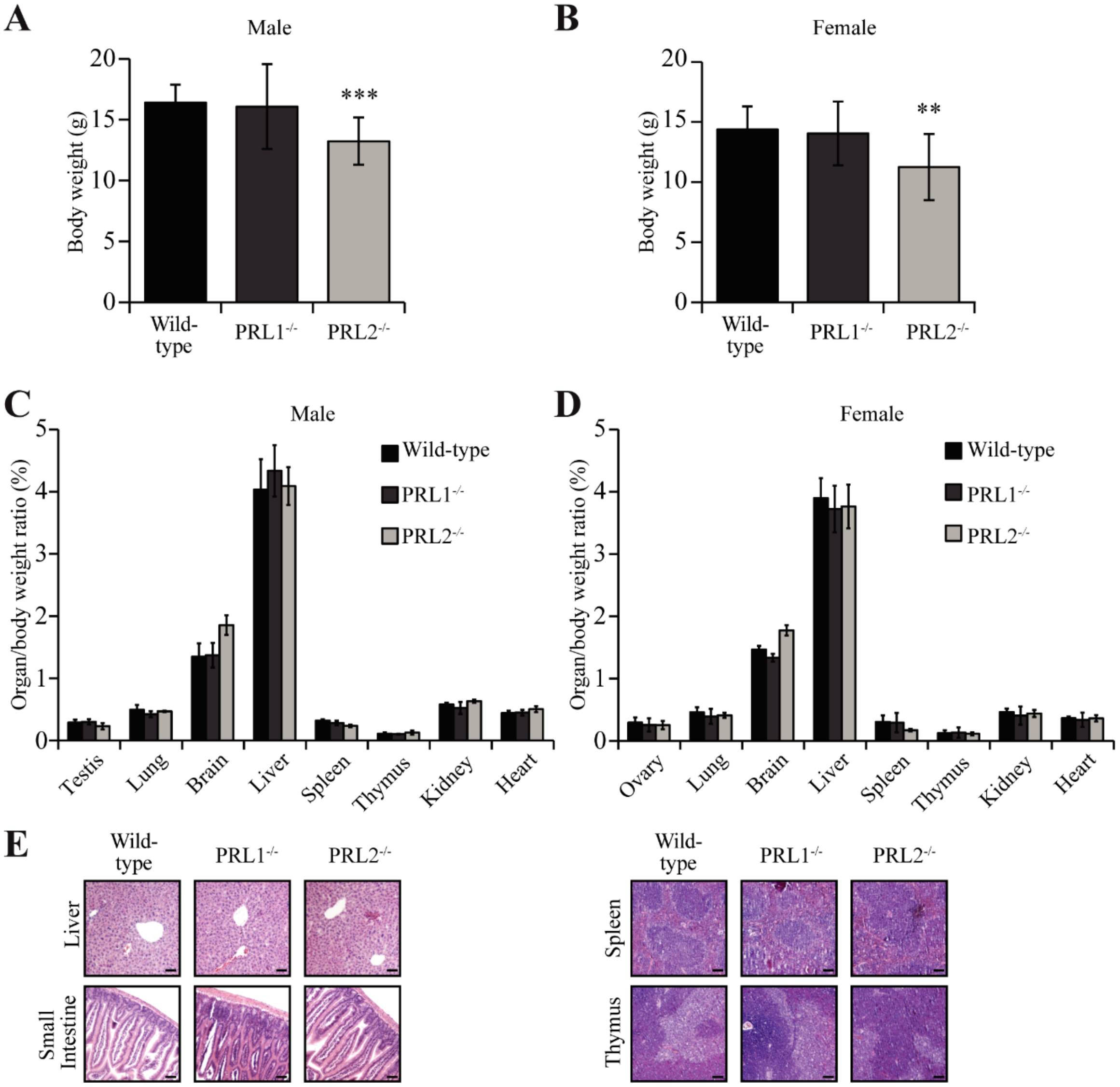

Supplemental Figure 3. Characterization of PRL1$^{-/-}$ and PRL2$^{-/-}$ mice. A-B. Body weights of either male (A) or female (B) wild-type, PRL1$^{-/-}$ and PRL2$^{-/-}$ mice at 4-week-old. C-D. The organ/body weight ratio of either male (C) or female (D) wild-type, PRL1$^{-/-}$ and PRL2$^{-/-}$ mice at 4-week-old. E. H&E staining of different organs from wild-type, PRL1$^{-/-}$ and PRL2$^{-/-}$ mice at 4-week-old. Scale bar = 50 µm.

Supplemental Figure 4

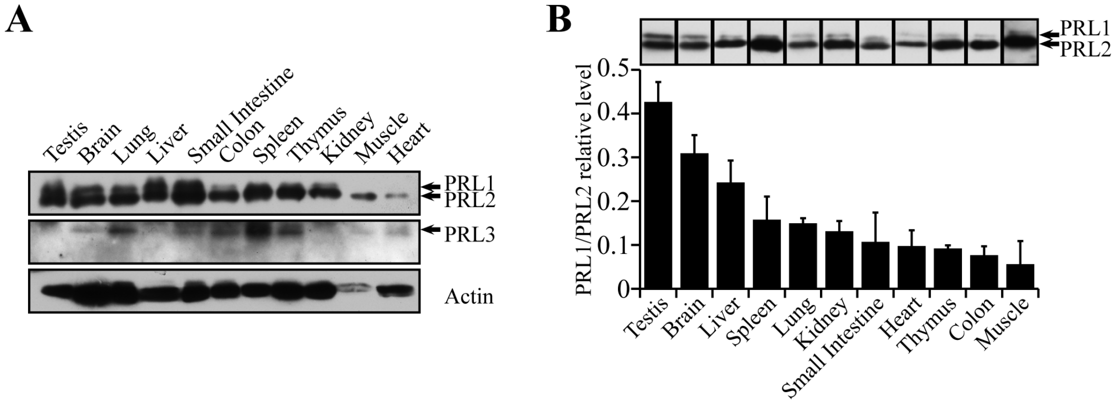

Supplemental Figure 4. PRL1, PRL2 and PRL3 expression in different organs. A. PRL1 and PRL2 are expressing ubiquitously in different organs, while PRL3 is only expressing in limited organs. B. Western blots of PRL1/PRL2 using PRL1/2 antibody (top panel) and the quantification of relative PRL1/PRL2 level (bottom panel) in different organs.

Supplemental Figure 5

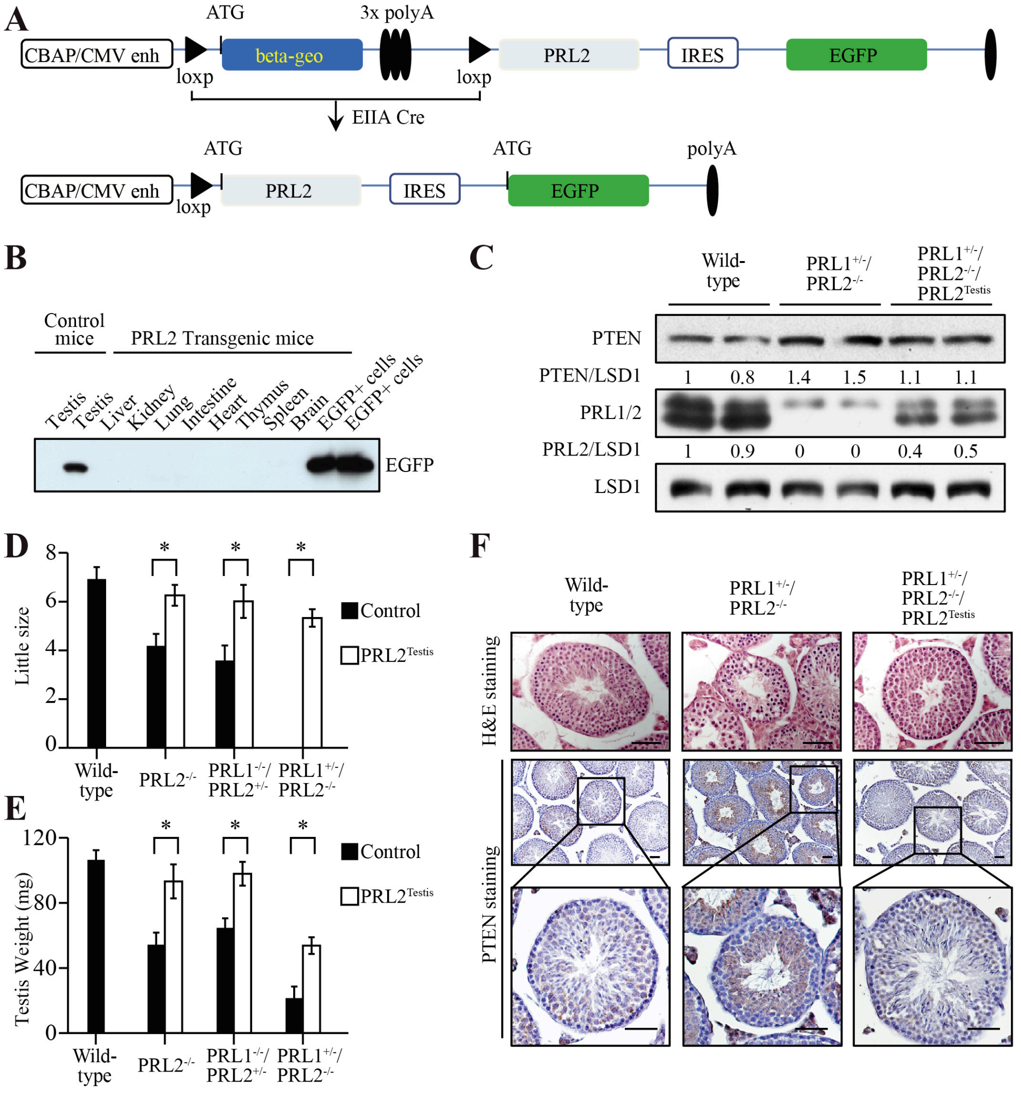

Supplemental Figure 5. Re-expressing PRL2 rescued PRL1 deletion-induced additional phenotypes in testis. A. Strategy to generate conditional PRL2 transgenic mice. B. Western blot indicates that the PRL2 transgenic mice only have PRL2 overeexpression in testis. C. Re-expression 50% of endogenous PRL2 by transgenic mice significantly restored PTEN level of PRL1$^{+/-}$/PRL2$^{-/-}$ mice to Wild-type level. D. Re-expressing PRL2 rescued reproductive ability in PRL2$^{-/-}$, PRL1$^{-/-}$/PRL2$^{+/-}$ and PRL1$^{+/-}$/PRL2$^{-/-}$ mice. E. Re-expressing PRL2 rescued testicular atrophy phenotype in PRL2$^{-/-}$, PRL1$^{-/-}$/PRL2$^{+/-}$ and PRL1$^{+/-}$/PRL2$^{-/-}$ mice. F. Re-expressing PRL2 rescued the histological abnormality (top

panel) and PTEN level (middle and bottom panels) in seminiferous tubules of PRL1$^{+/-}$/PRL2$^{-/-}$ mice.

Supplemental Figure 6

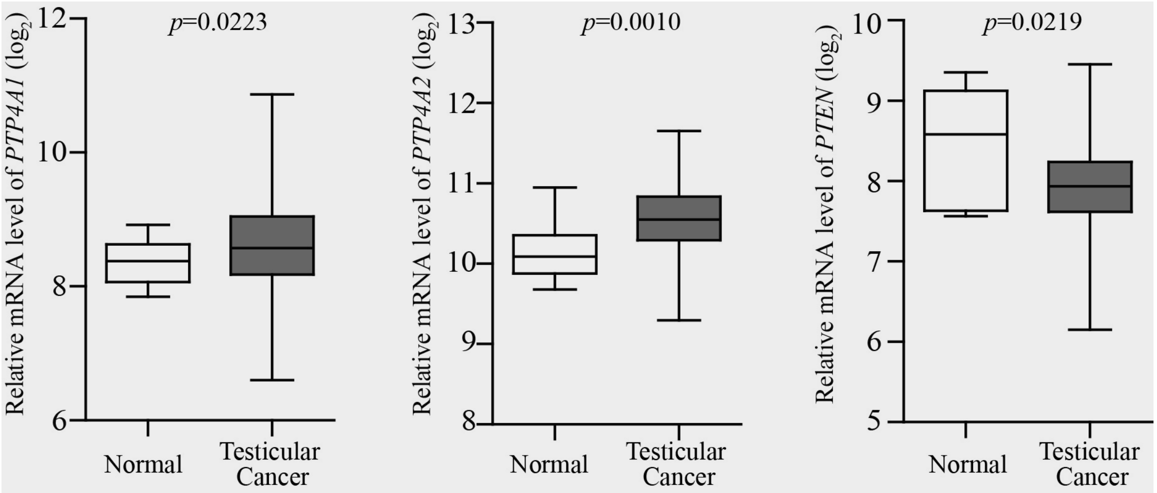

Supplemental Figure 6. PRL1, PRL2 and PTEN mRNA expression in testicular cancer patients. Differential expression of PRL1 (left panel), PRL2 (middle panel) or PTEN (right panel) mRNA between normal testis samples (n=13) and testicular cancer patient samples (n=184).

Supplemental Table 1. Total PRL1 and PRL2 level in different organs of mice with different genotypes.

	Testis	Brain	Liver	Spleen	Lung	Kidney	Small intestine	Heart	Thymus	Colon	Muscle
Wild-type	100%	100%	100%	100%	100%	100%	100%	100%	100%	100%	100%
$PRL1^{-/-}$	70%	76%	80%	86%	87%	88%	90%	91%	92%	93%	95%
$PRL2^{-/-}$	30%	24%	20%	14%	13%	12%	10%	9%	8%	7%	5%
$PRL1^{-/-}/PRL2^{+/-}$	35%	38%	40%	43%	43%	44%	45%	46%	46%	46%	47%
$PRL1^{+/-}/PRL2^{-/-}$	15%	12%	10%	7%	7%	6%	5%	4%	4%	4%	3%
